# Supplementary figures and images for: STK-12 acts as a transcriptional brake to control the expression of cellulase-encoding genes in Neurospora crassa
Source: PLoS Genet. 2019 Nov 25;15(11):e1008510. doi: 10.1371/journal.pgen.1008510 (PMC6901240; doi:10.1371/journal.pgen.1008510)

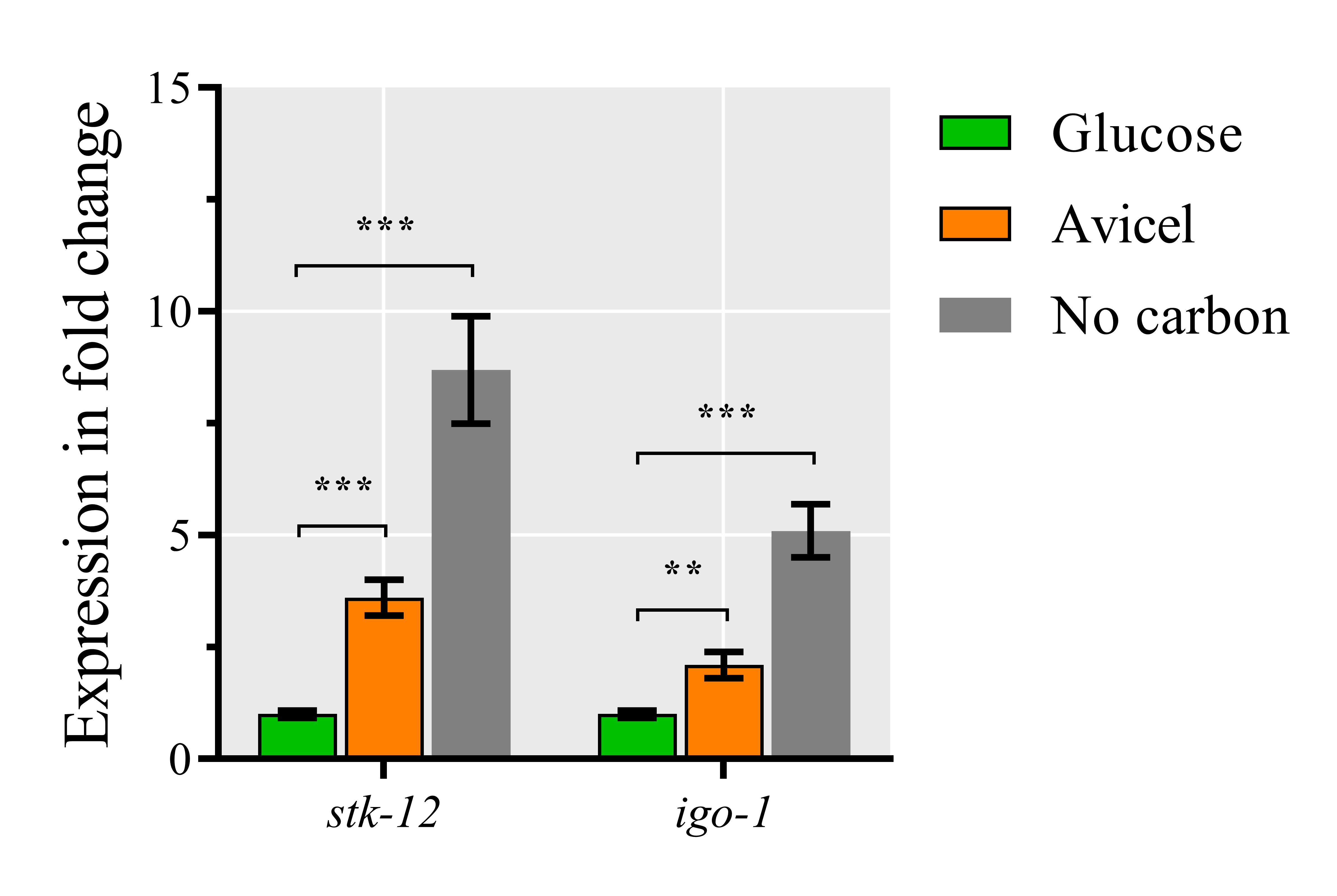

Supplement: S1 Fig — Gene transcript levels were normalized to 1 when induced with 2% (w/v) glucose. Actin (NCU04173) was used as the control. Transcript abundance was evaluated by quantitative real-time PCR. Values represent means of at least three biological replicates. Asterisks indicate significant differences from control (**, P < 0.01; ***, P < 0.001) based on two-tailed Student’s t-test. (JPG) [file pgen.1008510.s001.jpg]

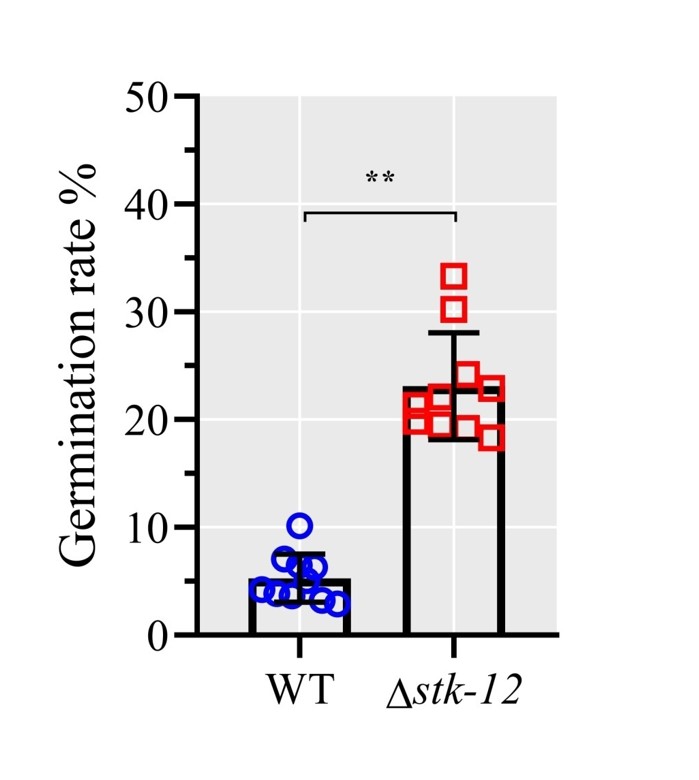

Supplement: S2 Fig — Conidia of Δstk-12 and WT were separately inoculated into 100 mL 2% w/v Avicel VMM at 106 conidia/mL and grown at 25°C in constant light with shaking (200 rpm). Germination rate was recorded at 6 h after inoculation. Circles indicate values of individual biological replicates. Error bars show standard deviation. Statistical significance was determined using two-tailed Student’s t-test (**, P < 0.01). (JPG) [file pgen.1008510.s002.jpg]

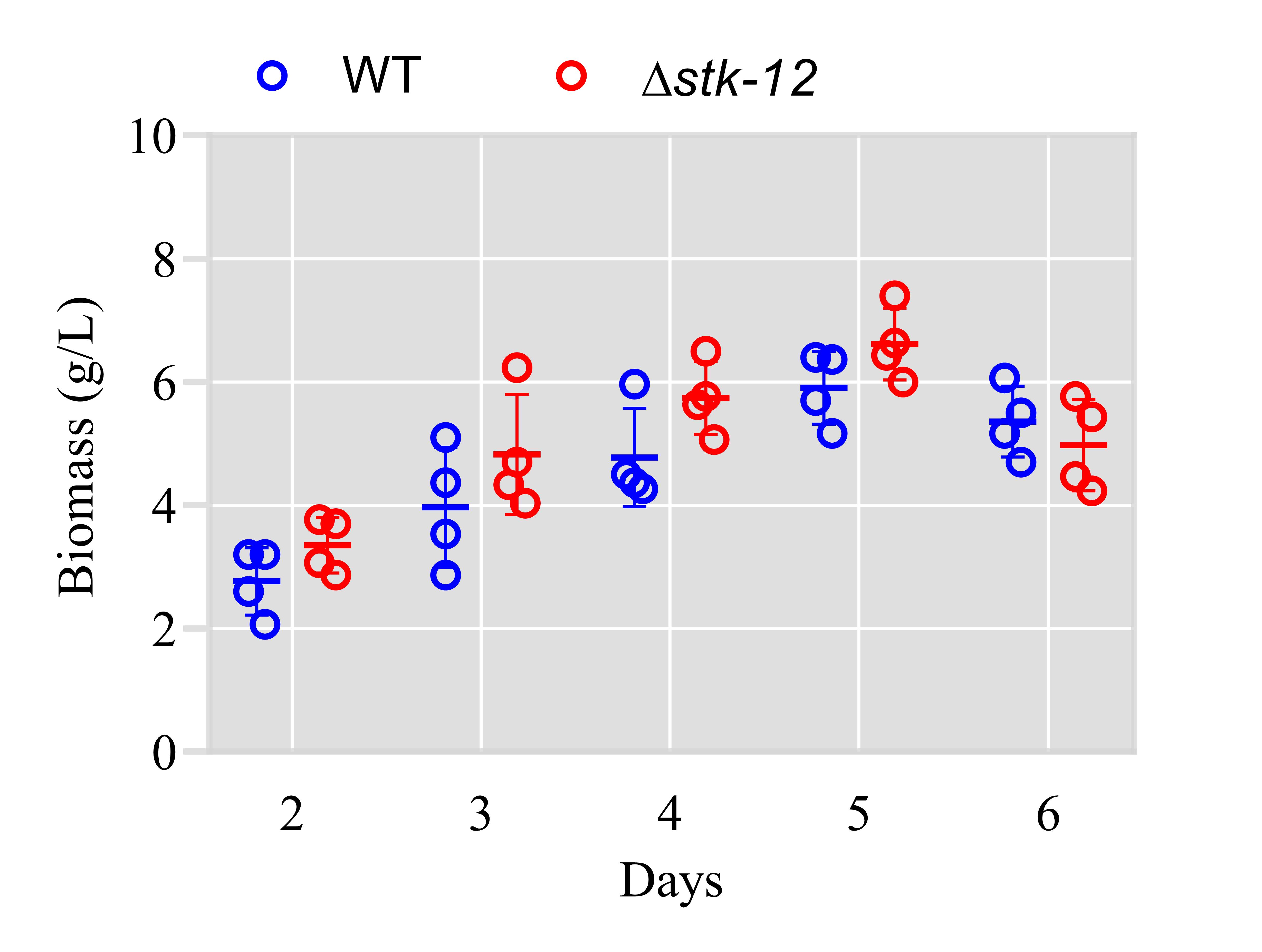

Supplement: S3 Fig — Conidia from Δstk-12 and wild type (WT) strains were inoculated into Avicel medium, respectively, and batch cultured for 6 days. The biomass accumulation was measured. Values represent the means of at least four replicates, error bars show standard deviation. (JPG) [file pgen.1008510.s003.jpg]

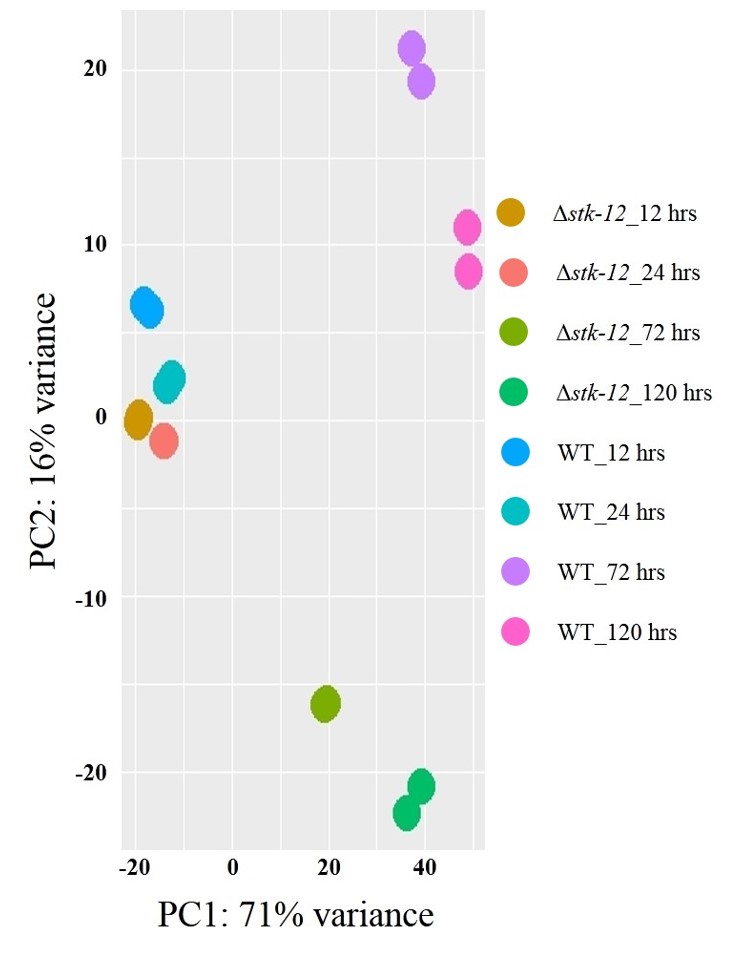

Supplement: S4 Fig — (JPG) [file pgen.1008510.s004.jpg]

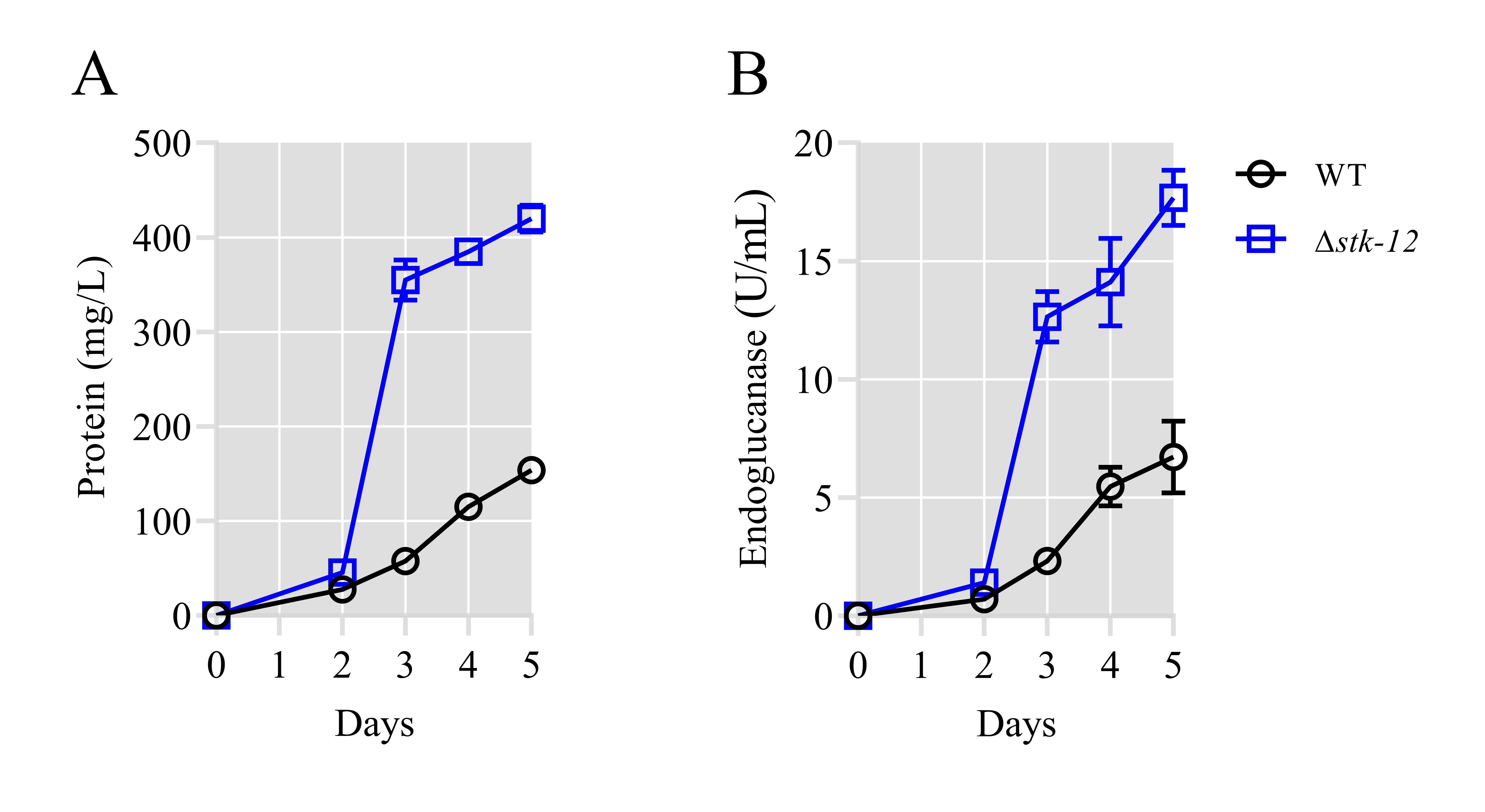

Supplement: S5 Fig — Total extracellular protein concentration (A) and endoglucanase activity (B). Values represent the means of at least three replicates, error bars show standard deviation. (JPG) [file pgen.1008510.s005.jpg]

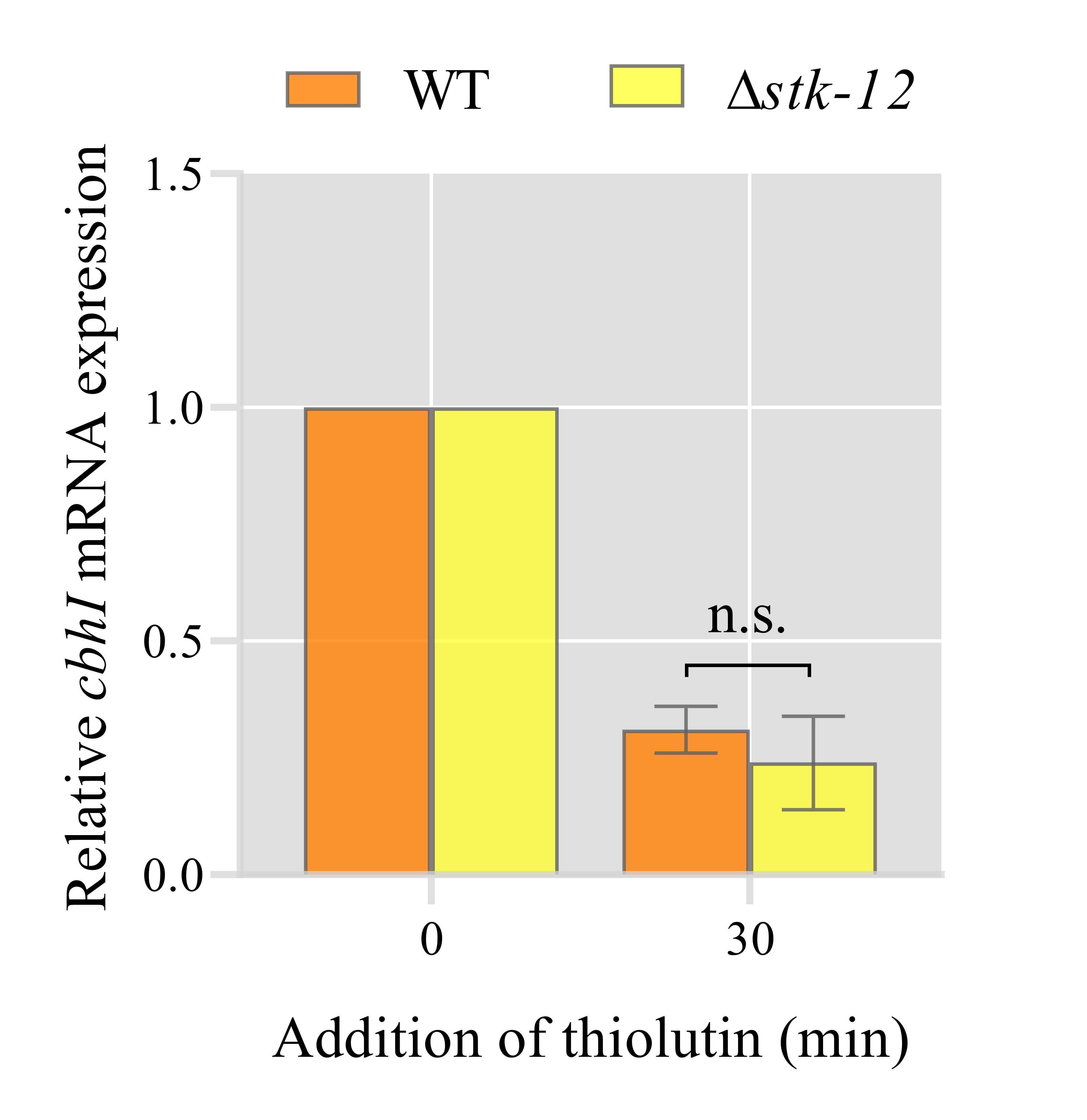

Supplement: S6 Fig — The decay of the cbh-1 mRNAs in the WT and stk-12 mutant is shown at the indicated time points after addition of thiolutin. Conidia from Δstk-12 and wild type (WT) strains were inoculated into Avicel medium, respectively, and batch cultured for 1 day. And then, thiolutin was added to a final concentration of 12 μg/mL to stop transcription. CBH-1 mRNA levels were measured by RT-qPCR and the levels of mature 26S rRNA was used as the internal control. (JPG) [file pgen.1008510.s006.jpg]

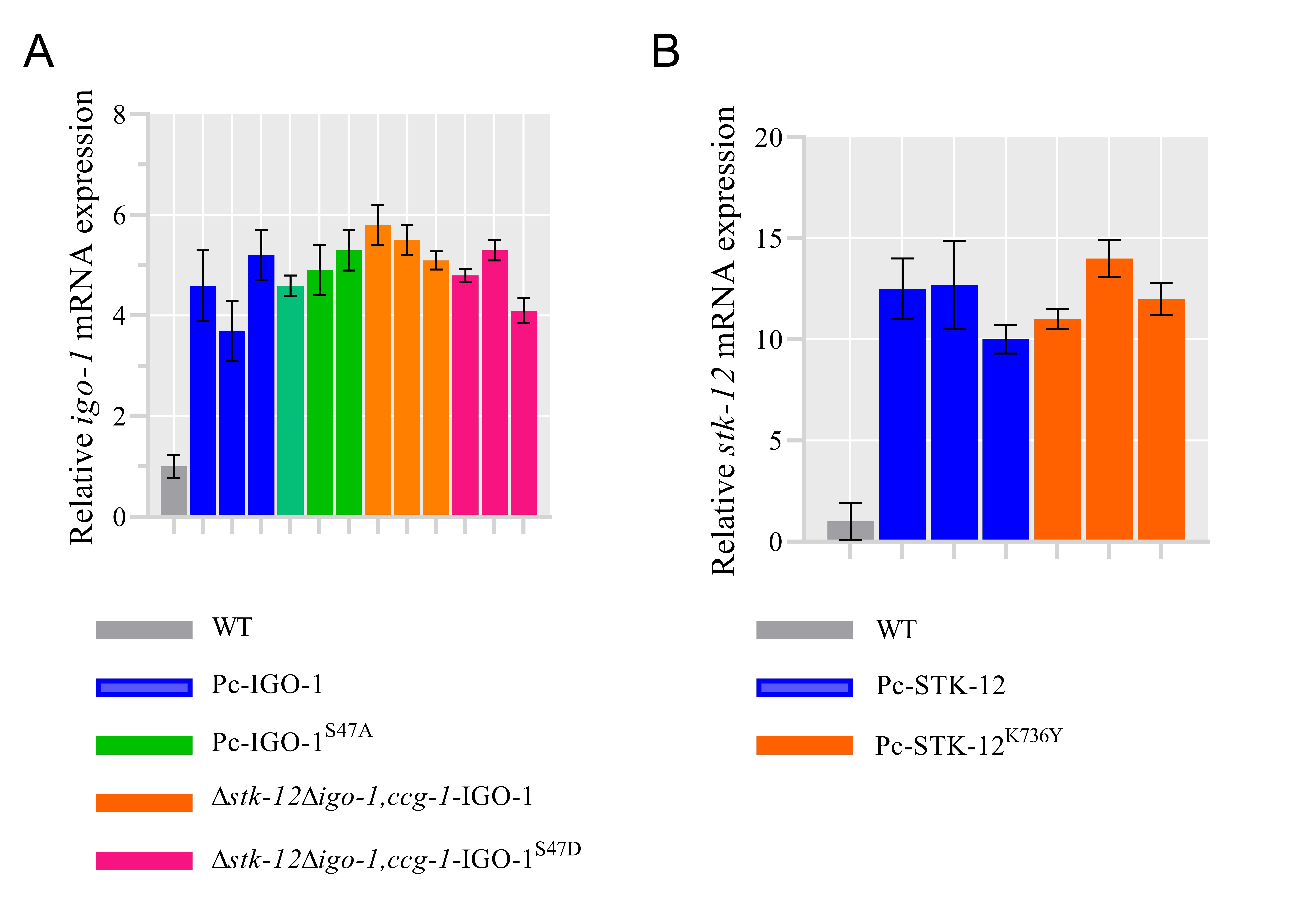

Supplement: S7 Fig — All strains were grown in MM for 16 h, and then transferred into Avicel medium for an additional 4 h. Gene expression levels were measured by RT-qPCR using actin as a control and normalized against the tested gene, igo-1 (A) or stk-12 (B), in WT strain. (JPG) [file pgen.1008510.s007.jpg]

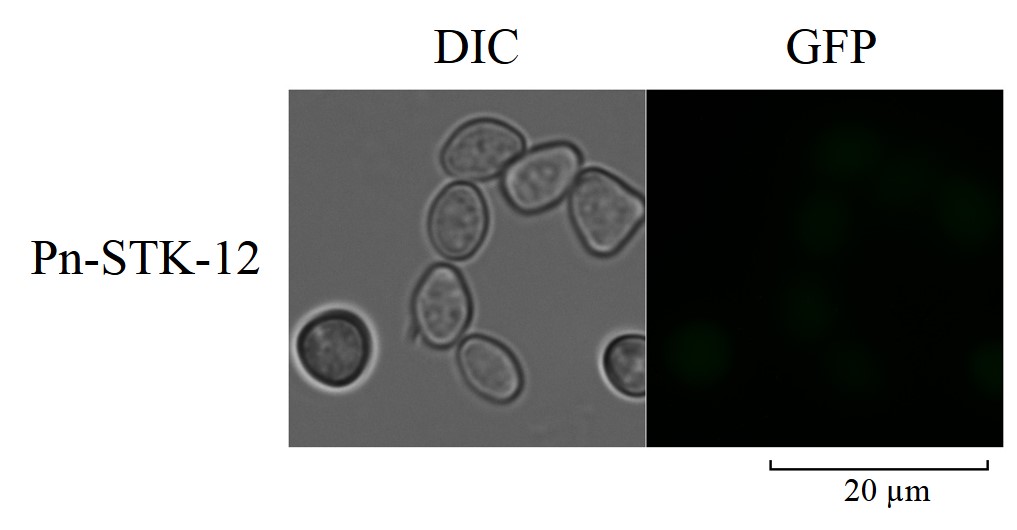

Supplement: S8 Fig — The strain with stk-12 under control of the native promoter (Pn-STK-12) was grown on MM plates for 5 days. Scale bar = 20 μm. (JPG) [file pgen.1008510.s008.jpg]

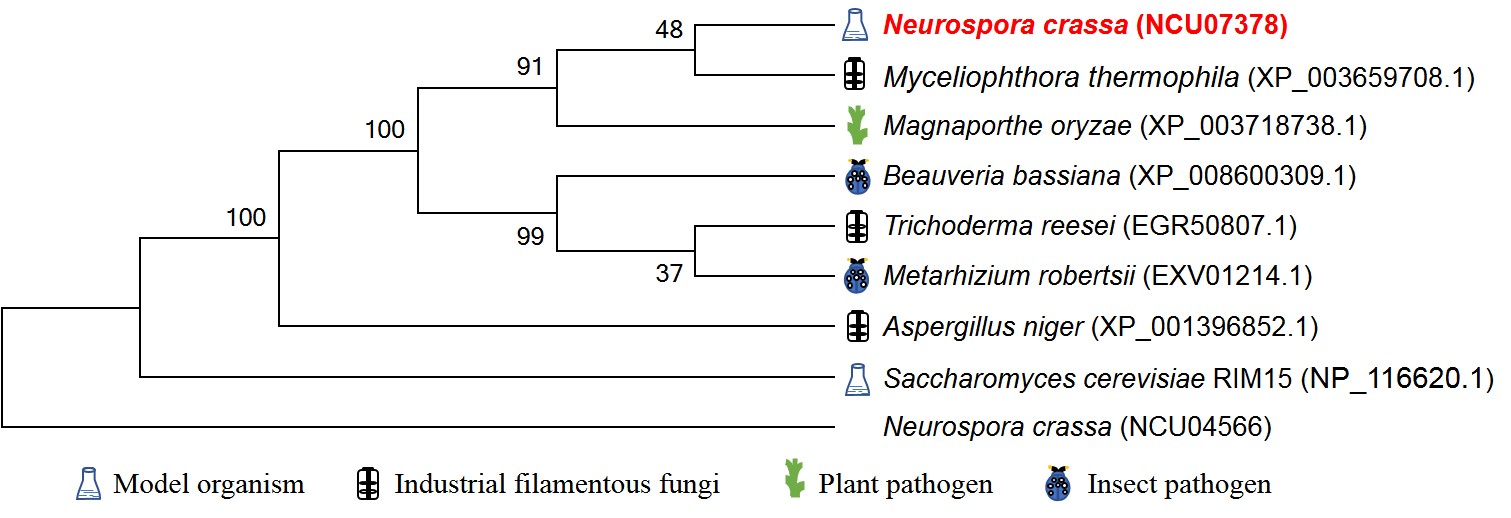

Supplement: S9 Fig — Bootstrap values are adjacent to each internal node (% of 1,000 bootstrap replicates). NCU04566 (PRK-10; SNF 1 homolog) from Neurospora crassa was used as outgroup. (JPG) [file pgen.1008510.s009.jpg]

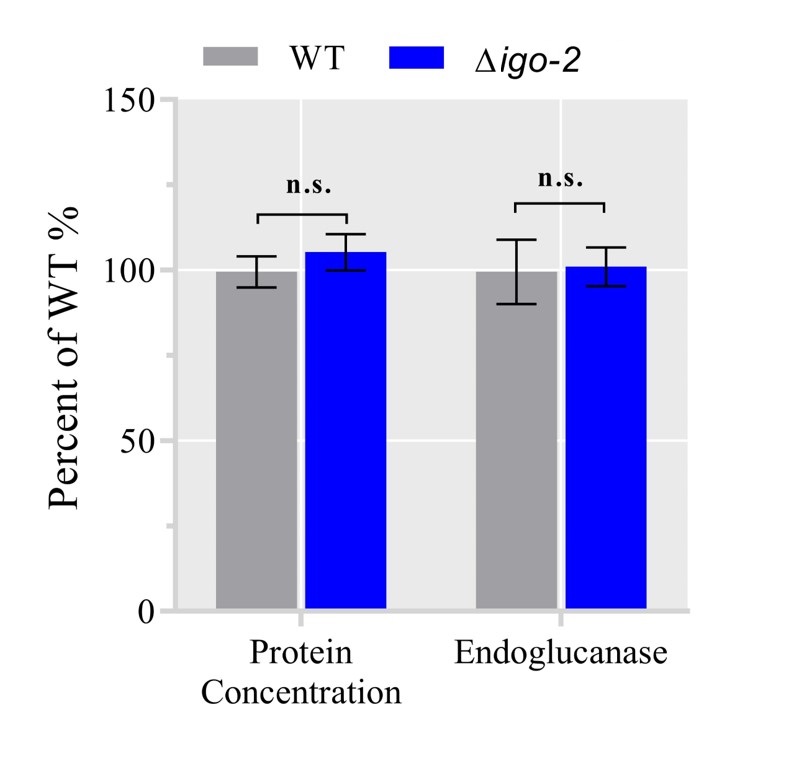

Supplement: S10 Fig — Avicel broth cultures were inoculated with conidia and grown for 5 days. Total extracellular protein concentration and endoglucanase activity of culture broth were measured and are expressed as a percentage of those in WT. Values represent means of at least three biological replicates, error bars show standard deviation. Statistical significance was determined by two-tailed Student’s t-test (n.s., not significant). (JPG) [file pgen.1008510.s010.jpg]

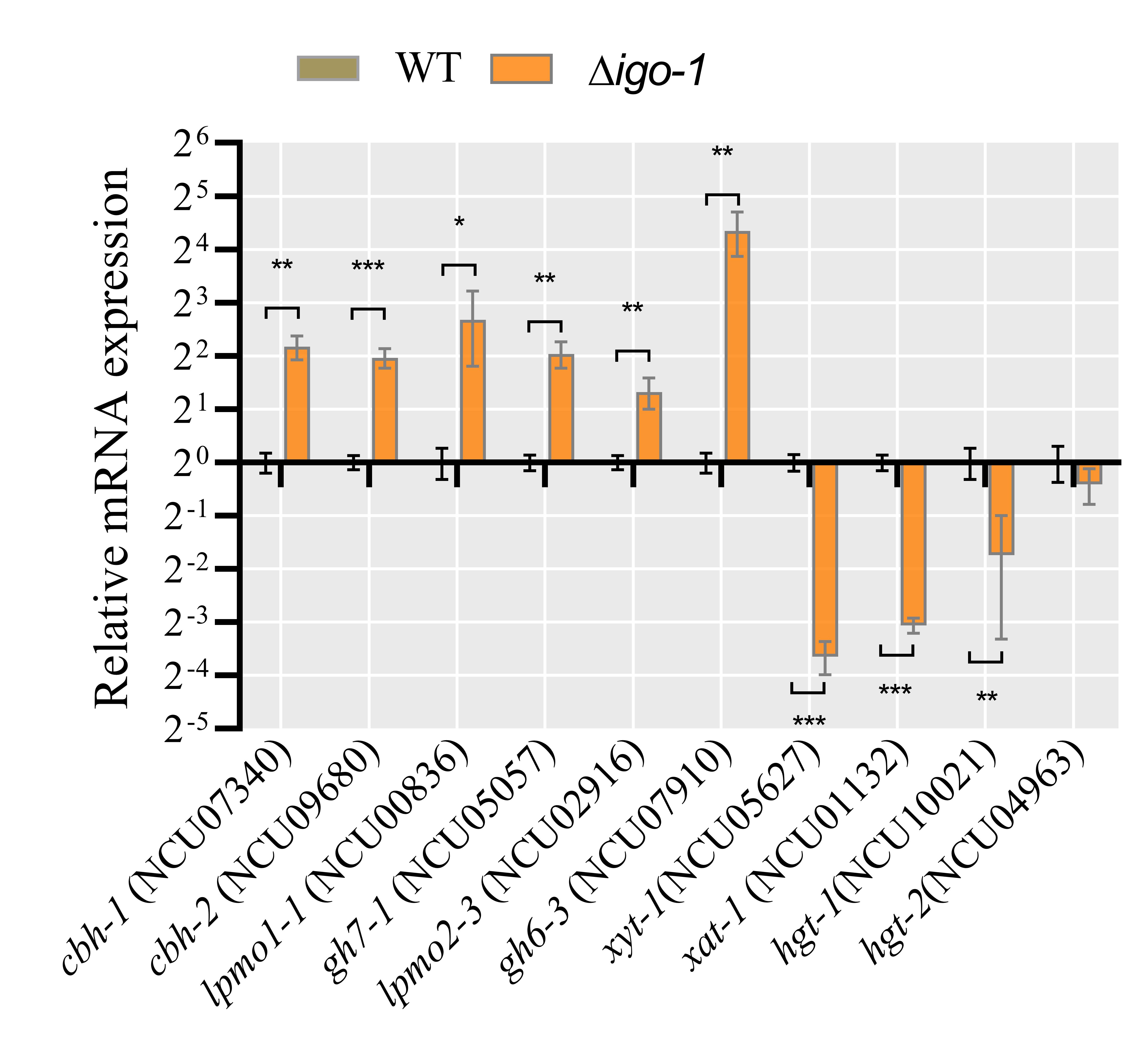

Supplement: S11 Fig — After Δigo-1 and WT conidia were grown on Avicel for 3 days, transcript abundance of indicated genes was evaluated by quantitative real-time PCR. Values represent means of at least three biological replicates, error bars show standard deviation. Statistical significance was determined by two-tailed Student’s t-test (*, P<0.05; **, P < 0.01; ***, P < 0.001, n. s., not significant). (JPG) [file pgen.1008510.s011.jpg]

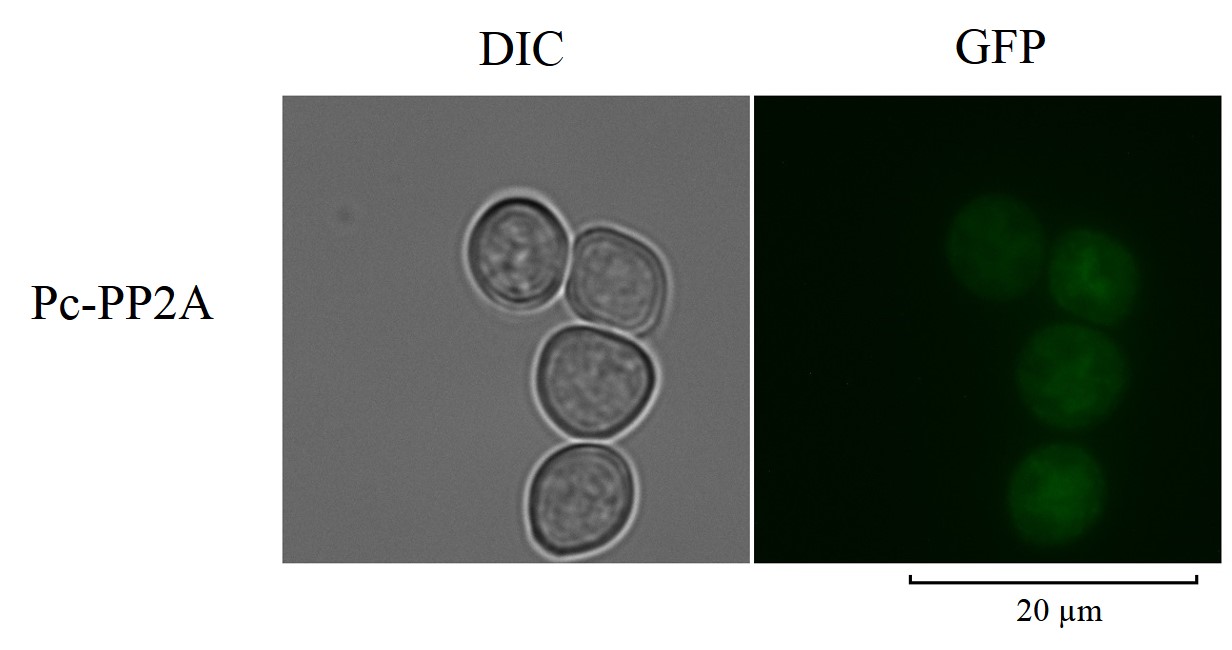

Supplement: S12 Fig — Strain with NCU06563 under control of ccg-1 promoter was grown on MM plates for 5 days. Location of PP2A was monitored by GFP fluorescence. Scale bar = 20 μm. (JPG) [file pgen.1008510.s012.jpg]

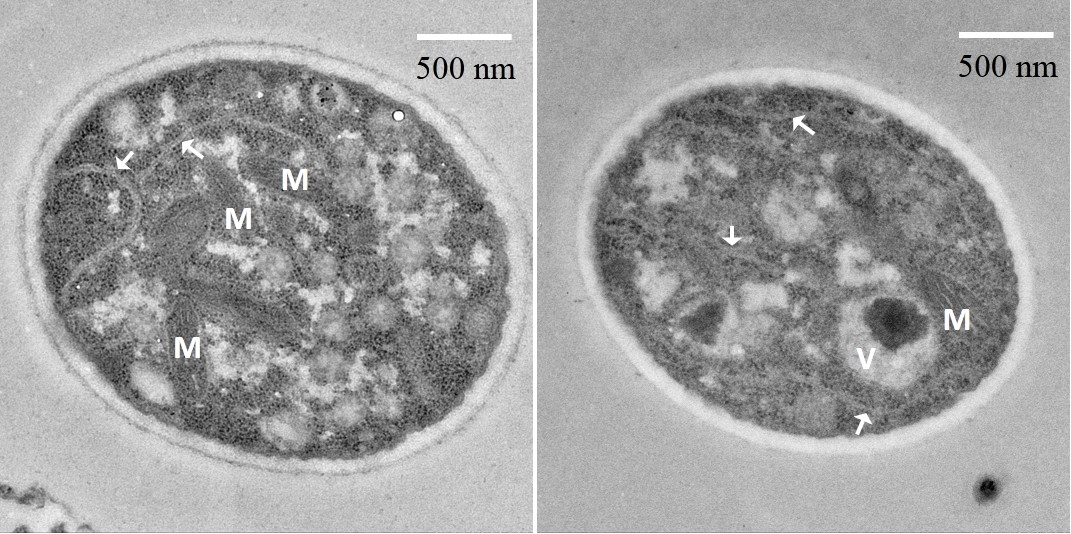

Supplement: S13 Fig — Mycelia were collected and prepared for transmission electron microscopy. White arrows indicate endoplasmic reticulum. M, mitochondrion; V, vacuole. (TIF) [file pgen.1008510.s013.tif]
